# Insulin–cobalt core–shell nanoparticles for receptor-targeted bioimaging and diabetic wound healing†

**DOI:** 10.1039/d3ra01473h

**Published:** 2023-07-06

**Authors:** Deepinder Sharda, Diptiman Choudhury

**Affiliations:** a School of Chemistry and Biochemistry, Thapar Institute of Engineering and Technology Patiala 147004 Punjab India diptiman@thapar.edu +91-8196949843; b Thapar Institute of Engineering and Technology-Virginia Tech (USA) Center of Excellence in Emerging Materials, Thapar Institute of Engineering and Technology Patiala Punjab-147004 India

## Abstract

Diabetic wounds represent a major issue in medical care and need advanced therapeutic and tissue imaging systems for better management. The utilization of nano-formulations involving proteins like insulin and metal ions plays significant roles in controlling wound outcomes by decreasing inflammation or reducing microbial load. This work reports the easy one-pot synthesis of extremely stable, biocompatible, and highly fluorescent insulin–cobalt core–shell nanoparticles (ICoNPs) with enhanced quantum yield for their highly specific receptor-targeted bioimaging and normal and diabetic wound healing *in vitro* (HEKa cell line). The particles were characterized using physicochemical properties, biocompatibility, and wound healing applications. FTIR bands at 670.35 cm^−1^, 849.79, and 973.73 indicating the Co–O bending, CoO–OH bond, and Co–OH bending, respectively, confirm the protein–metal interactions, which is further supported by the Raman spectra. *In silico* studies indicate the presence of cobalt binding sites on the insulin chain B at 8 GLY, 9 SER, and 10 HIS positions. The particles exhibit a magnificent loading efficiency of 89.48 ± 0.049% and excellent release properties (86.54 ± 2.15% within 24 h). Further, based on fluorescent properties, the recovery process can be monitored under an appropriate setup, and the binding of ICoNPs to insulin receptors was confirmed by bioimaging. This work helps synthesize effective therapeutics with numerous wound-healing promoting and monitoring applications.

## Abstract 2

Diabetic wounds represent a major issue in medical care and need advanced therapeutic and tissue imaging systems for better management.

## Graphical abstract

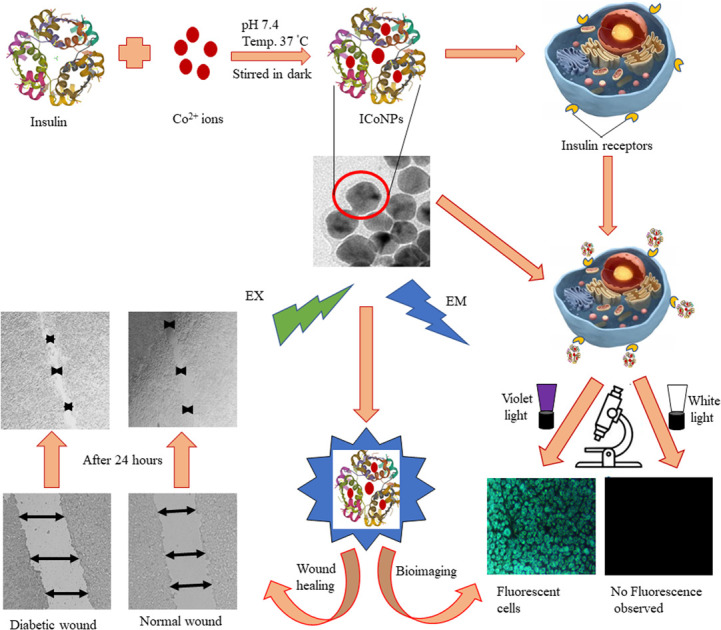

## 1. Introduction

Wound healing is a complex process involving a sequential overlapping cascade of events that comes into action in response to some external chemical or physical stimuli and eventually causes healing by restoring lost tissue.^1^ The healing process is categorized into four phases, hemostasis, inflammation, proliferation, and remodeling.^2^ The hemostasis is initiated by clot formation due to platelets' activation, which prevents microbial infestation and promotes matrix organization. In proliferation, the accumulation of cells, connective tissue, growth factors, and angiogenesis factors across the wound occurs. The remodeling involves the resynthesis of the extracellular matrix to maintain the balance between the death of existing cells and the formation of new cells.^3,4^ However, the progress monitoring of recovery of wounds always remains a significant challenge. In some instances, normal healing gets disrupted. It gets arrested in one of the phases due to the loss of balance in the physiological mechanism of healing due to infection, chronic irritation, trauma, the persistence of microbes or other foreign bodies, and ischemia, making the wound a chronic one^5–7^ which can be prevented by keeping the wound moist, removing the dead tissues, covering the injury to avoid bacterial infection, and removing the excess tissue fluid.^8^

Apart from these issues, the major problem for delayed healing is diabetes mellitus or long-term hyperglycemia, which alters the secretion of cytokines, making wound healing cumbersome.^9^ In diabetic conditions, there is the prolonged proinflammatory phase due to the persistent release of proteases, proinflammatory cytokines, and reactive oxygen species and the delayed anti-inflammatory phase because of the low secretion of anti-inflammatory cytokines.^10^ Further, the transport of nutrients to the wound site is prevented by atherosclerosis caused by diabetes.^11^ It also results in the dysfunction of endothelial cells due to vasodilation induced by pressure^12^ and disrupts the processes essential for re-epithelialization; the proliferation of keratinocytes and fibroblasts, synthesis of proteins, and cell migration.^13–15^ Further, it impairs the body's immune response, making the wounds prone to infection and leading to damaged structural components of the extracellular matrix.^7^ It also causes free radical damage due to the reduced activity of certain antioxidant enzymes such as glutathione peroxidase and superoxide dismutase.^16^ Various advanced bandages are developed for treating diabetic wound healing and overcoming the issues faced in effective healing.^17,18^

Recent advancements in nanotechnology, such as specific physicochemical properties and small size, allow the intracellular delivery of different drugs and biomolecules, protect them from degradation, increase the penetration ability of drugs into the wound, allow the topical application, and enhance the half-life of this agents.^19,20^ It results in a decline in repetitive drug application, thus lowering the cost and making them cost-effective.^21,22^ The metallic nanoparticles possess a high surface-to-volume ratio, high stability, biocompatibility, safety, and economical rates.^22,23^ Several gold, silver, copper, zinc, and cobalt metallic nanoparticles were prepared to study wound healing activities *in vitro* and *in vivo* models.^24^ Protein-based nanoparticles are gaining attention due to their high biodegradability, easily metabolizable nature, and the possibility to alter or modify their surface for the drugs to attach efficiently. The proteins can be of plant or animal origin, such as bovine serum albumin,^25^ insulin,^26^ transferrin,^27^ lactoferrin, and so on, for nano-formulation synthesis.^28,29^ The selection of nanoparticles in treating wounds is based on three parameters; antimicrobial action, role as a delivery agent, and role in the repair process.^30^

The use of insulin protein is increasing due to the enormous presence of insulin receptors on the membranes of all mammalian cells, and there is a significant variation in the number of receptors from just 40 in erythrocytes to 200–300 × 10^3^ for adipocytes and hepatocytes; however, the number is much higher in cancerous cells compared to normal cells.^26,31^ It plays a crucial role as a growth factor that facilitates chemotaxis and pinocytosis or phagocytosis by macrophages, promotes the secretion of cytokines critical for inflammation, and helps in re-epithelialization, essential for wound healing. It works by transitioning proinflammatory cytokines into anti-inflammatory cytokines and promotes wound repair and regeneration.^32,33^ The nano-formulations involving insulin as a protein template exhibit promising applications in bioimaging, super-resolution microscopy, normal wound healing, and diabetic wound healing.^32–35^ The metal ions used earlier to form different formulations with insulin include silver, copper, zinc, nickel, *etc.*^31^ Apart from metal–insulin nanoparticles, other nano-formulations involving insulin as a wound-healing agent have been synthesized. Insulin-containing chitosan nanoparticles were synthesized to stimulate inflammatory cell proliferation, angiogenesis, and wound healing in diabetic rats.^36^ Lee *et al.* reported accelerated diabetic wound repair by promoting epithelialization using core–shell insulin-loaded scaffolds.^37^ The synthesis of human keratin conjugated insulin was reported by Li *et al.* for their role in full-thickness wound repair in rats by promoting cellular migration.^38^ Clinical studies were also performed to confirm the role of insulin further. The insulin injection was used to study the effect on systemic blood glucose levels and healing of diabetic foot ulcers in patients and found that insulin promotes the formation of vessels and granulation tissue.^39^ The impact of topical insulin was studied in 110 patients having diabetic foot ulcers using a double-blind placebo-controlled trial and found that patients treated with insulin gauze dressings had a wound diameter of 2.46 ± 0.57 cm after two weeks, while patients treated with saline gauze had a diameter of 3.90 ± 0.76 cm indicating the significant effect of insulin.^40^ Similarly, a mucoadhesive liposomal gel of insulin was explored on patients with chronic wounds in different body parts, demonstrating a more significant impact on test groups than control groups.^41^

The cobalt ions also help in wound healing and act as an alternative to hypoxia-mimicking agents. It inhibits the activity of PHDs (prolyl 4-hydroxylases), artificially stabilizes the HIF-1α (hypoxia-inducible factor-1α) under normal conditions, and activates the HIF pathway,^42^ allowing the formation of the HIF complex, which gets translocated to the nucleus and causes upregulation in the expression of genes such as VEGF, which are responsible for adaptation to hypoxia.^43^ As a result, it promotes angiogenesis during the tissue regeneration process.^44^ It was also found that incorporating cobalt ions in titanium-based bone implants enhances their antibacterial activity, indicating the role of cobalt as an antimicrobial agent.^45^ Further, the release of cobalt ions from the bioactive glass increases the VEGF protein secretion and osteogenic expression of genes by human bone marrow stromal cells.^46,47^ Moreover, if the cobalt ions are delivered as cobalt chloride, they enhance the secretion of VEGF by endothelial cells,^48^ fibroblasts,^49^ and human mesenchymal stem cells.^50^ The synthesis of silicate glass fibers for delivering the cobalt ions was done for their chronic wound healing activity by activating the HIF pathway and enhancing the expression of angiogenic genes.^51^ Similarly, a bioactive glass was made using the sol–gel method and doped with two different metal ions, including silver and cobalt, for their antibacterial activity in the case of wound healing applications.^52^

In this work, we are reporting the synthesis of novel protein-coated metal core–shell nanoparticles for their *in vitro* normal and diabetic wound healing and bioimaging application for the first time. Here, by using one-pot synthesis, we have prepared insulin–cobalt core–shell nanoparticles to check the synergistic effect of insulin and cobalt for treating wounds when applied in the form of particles in the nano range. The effective and efficient healing of wounds is essential for maintaining a person's life quality. Still, in some instances, the absence of proper internal wound monitoring makes the process even more complex, increasing the cost of treatment and putting risk to a patient's life. To overcome such challenges, the utilization of developed nanoparticles with dual healing and bioimaging capabilities offers a promising solution for enhanced wound management.

## 2. Materials and methods

### 2.1 Chemicals and cell line

The metal salt, cobalt chloride (CoCl_2_·6H_2_O), tryptophane, formaldehyde, HCl, and NaOH were of analytical grade and were purchased from Sigma Aldrich, India. Recombinant human insulin was purchased from Elli Lilly, India. For cell culture, DMEM cell culture media, Fetal Bovine Serum (FBS), 100× penicillin–streptomycin, and phosphate buffer solution (PBS) having pH 7.4 were purchased from HiMedia, India. Human Primary Epithelial Keratinocytes (HEKa cells) ATCC-PCS-200-011 were procured from Himedia, India, cultured, maintained, and treated in DMEM containing 5% FBS at 37 °C and 5% CO_2_.

### 2.2 Synthesis of insulin-protected cobalt nanoparticles

At first, the insulin-protected core–shell cobalt nanoparticles were prepared by following a previously reported one-pot method in which the pH of the insulin solution having a final concentration of 1.82 μM, was adjusted to 10.5 using a NaOH solution (0.1 N) and kept in the dark. Afterward, a salt solution (CoCl_2_·6H_2_O) of the same molarity was prepared, followed by mixing two solutions in 1 : 1 by volume, which was further followed by adjustment of the pH of the final solution to a physiological pH of 7.4, using HCl (0.1 N). The resulting solution was kept in the incubator at slow stirring (240 rpm) for 24 h at 37 °C.^53–55^ The final solution was dialyzed using a 10 kDa cut-off dialysis membrane, stored at 4 °C, and used further for characterization. Thus, produced insulin–cobalt nanoparticles were termed as ICoNPs.

### 2.3 Study of particle size, morphology, and elemental analysis

The DLS (dynamic light scattering) was done to determine the hydrodynamic size of ICoNPs formed using a Malvern DLS-Zeta size analyzer. Thereafter, to find out the morphology of insulin-linked cobalt nanoparticles, Scanning Electron Microscopy (SEM JEOL, JSM-6300) and High-Resolution Transmission Electron Microscopy (HRTEM) (Talos F200S G2, Thermo Scientific) were used. For this, the samples were centrifuged at 240 rpm for 15 minutes, then thoroughly washed the pellet to remove unbound metal salt or impurities associated with the sample. Furthermore, the sample pellet was investigated to determine the percentage of the elements present by the Energy dispersive X-ray Spectrometer (EDS) (Bruker QUANTAX 200).

### 2.4 *In silico* studies

To determine the potential binding sites for different transition metal ions with the desired protein, MIB (Metal Ion Binding), an online docking tool, can be used as a user-friendly and integrated approach for finding the residues in metal binding sites. For operating this online server, protein chains were required, which were extracted from the protein data bank (PDB). The first chain consists of the template protein (T) of length *m* containing metal-ion, and the second chain consists of query protein (S) of length *n*. These chains were then aligned properly so the metal ion binding protein template could be converted into the query protein structure after alignment. Several different parameters were followed to obtain the desired protein structures. The first and the most crucial parameter is the prediction of binding residues with the twelve transition metal ions, including Ni^2+^, Cu^2+^, Mg^2+^, Ca^2+^, Hg^2+^, Cd^2+^, Co^2+^, Zn^2+^, Fe^2+^, Mn^2+^, Cu^+^, and Fe^3+^. The second factor is that the polypeptide chain length in the protein structures should have 50 residues; otherwise, it will be excluded.^56^ The third parameter is the presence of at least two metal ion binding residues having the minimum possible distance between the PDB coordinates and the metal center.^57^ The fourth crucial parameter is the allotment of a binding score to the residue, which should be greater than a specified threshold value assigned and calculated using the root mean square deviation of C-alpha carbons of local structural alignment and BLOSUM62 substitution. If so, only then will there be binding between the residue and the particular transition metal ion. Further, the binding score is denoted as CI and depends upon the target protein's sequence along with the protein's structural conservation.^58,59^ Hence, the binding site of metal ions was determined using the bioinformatics tool.

In this manner, the binding site of a particular metal with that of a protein chain can be determined using the MIB tool. The PDB (Protein Data Bank) database was used to extract human insulin protein (PDB ID: 4EWW), a polypeptide with two distinct chains denoted as A and B, respectively. In the MIB tool, the Co^2+^ metal ion was independently docked with chains A and B of human insulin, followed by a comparison of the metal ion-binding score with that of the target protein.^60^

### 2.5 Study of cobalt–insulin interaction using spectroscopic techniques

To study the metal–protein interactions in the ICoNPs, FTIR was performed using an Agilent Cary 600 series Spectrophotometer. The potassium bromide (KBr) method was used to prepare the samples, and scanning was done from 400 cm^−1^ to 4000 cm^−1^.

Furthermore, the same samples were analyzed using Surface Enhanced Raman Scattering (SERS) Spectra to study the structural changes in insulin protein.^63^ All the samples were prepared on a silicon wafer ten minutes before the measurement. The samples were scanned from 500 cm^−1^ to 1800 cm^−1^. The LabRam Hr Evolution Horiba, equipped with a detector and microscope, was used to record the Raman spectra of insulin and ICoNPs at 785 nm.

In order to monitor the changes in the secondary structure of the protein molecule, CD (Circular Dichroism) is the most reliable technique. In addition to the above studies, CD studies were conducted to confirm the interaction between insulin and cobalt chloride salt solution (freshly prepared and after 1 month). We have performed the Circular Dichroism (CD) spectroscopy using the instrument Mos500 CD biologic. The CD studies were performed at 25 °C with 1 ml of sample and scanned in the 200–260 nm wavelength range for pure insulin and insulin cobalt nanoparticles using phosphate buffer (pH 7.4) as a solvent.

### 2.6 Drug-loading and release kinetics

To monitor the release kinetics, 1 ml of the ICoNPs was centrifuged for 15 min at 10 000 rpm. The Bradford reagent determined insulin concentrations from the supernatant and the pellet. Further, the insulin release rate was measured at specific time intervals for 40 hours. The absorption values (at 595 nm) were later plotted to note the trend of drug release using BSA standard curves.^61,62^ In addition, absorbance values were also measured for the same samples at 272 nm (excitation of tryptophan) to support the data further.

### 2.7 Absorption and fluorescence spectroscopic study

For obtaining the UV-visible absorbance, the UV-2600 spectrophotometer of Shimadzu was operated between 200–800 nm, and a 4000 μl quartz cuvette with a 1 cm path was used, and the absorbance for insulin, salt solution (CoCl_2_), and insulin linked cobalt core–shell nanoparticles (ICoNPs) were measured.

For measuring the fluorescence data of ICoNPs, Agilent technologies, a Cary Eclipse fluorescence spectrophotometer was used, which helps in determining the fluorescence intensity of protein-linked metal nanoparticles (ICoNPs), insulin, and CoCl_2_ solution, all having the same concentrations, at an excitation wavelength of 272 nm, coupled with an emission scan from 280 nm to 800 nm with excitation and emission slit of 10 mm.

In order to calculate and compare the percentage increase in intrinsic fluorescence intensity of both insulin and ICoNPs, the given eqn (a) was used.a
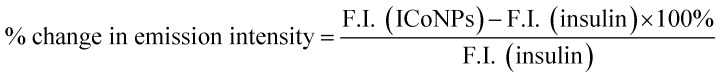


The standard tyrosine fluorescence quantum yield was used to calculate the quantum yield of insulin as well as insulin-protected cobalt nanoparticles (ICoNPs), and the given eqn (b) was used for further calculations.b
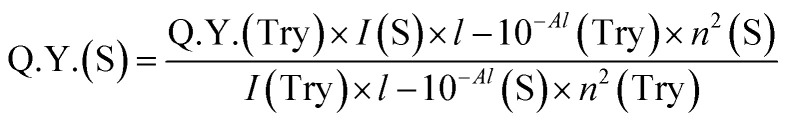
Here, Q.Y.is quantum yield; *I* is integrated emission intensity; *n* is the refractive index of solvent; *A* is the absorbance at excitation wavelength; *l* is the length of absorption cell; Tyr is tyrosine (reference), and S is sample.

### 2.8 Stoichiometry ratio of Co^2+^ ions : insulin protein

To understand the interactions between metal–ligand complexes and complex proteins, the stoichiometry of cobalt ions to insulin protein is calculated using the following eqn (c).c
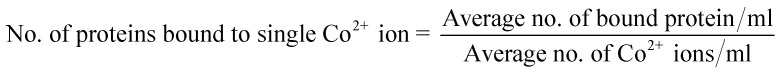


### 2.9 *In vitro* studies

#### 2.9.1 Cell viability

In order to determine the viability of cells in the presence of all samples, the HEKa cell line (Human Epidermal Keratinocytes, adult) was used, and MTT (3-(4,5-dimethylthiazol-2-yl)-2,5-diphenyltetrazolium bromide) assay was performed. In order to perform this assay, HEKa cells were seeded into 96 well plates with a density of 1 × 10^4^ cells per well and were kept in the incubator for the cells to grow and become 75–80% confluent with the regular addition of media. Once the plate became confluent, the cells were incubated with ICoNPs, insulin, CoCl_2,_ salt solution, and a mixture of insulin and CoCl_2_. To get three concordant readings, four different concentrations of each sample that are 1.5, 7.5, 30, and 60 μM are added to respective wells. Thereafter, the plate was kept in an incubator for a time-lapse of 24 h and at a temperature of 37 °C. After 24 h, the media was discarded, followed by the addition of new media, and MTT (2 mg ml^−1^ in 5% ethanol) was added into each well, and the plate was placed as such for 3 h in an incubator. The solution of MTT and media was removed from each well after 3 h, followed by the addition of 200 μl dimethyl sulfoxide (DMSO) to dissolve the formazan crystals. Finally, the absorbance was checked at a wavelength of 575 nm. To calculate the inhibition percentage, the equation used is as follows

d % inhibition = [1 − (*A*_t_/*A*_c_) × 100]%

Here *A*_t_ is the absorbance of the test substance, and *A*_c_ is the absorbance of the control solvent for each concentration.^64^

#### 2.9.2 Internalization study of nanoparticles into cells

STEM analysis was performed to study synthesized nanoparticles' interaction with human cells. To do the internalization studies HEKa cell line was used. The cells were grown in 35 mm plates and let to be confluent till 80–85% and then were treated with ICoNPs and incubated for 24 hours. After 24 hours, the cells were trypsinized and centrifuged, and the pellet was fixed using 2.5% glutaraldehyde in 0.03 M phosphate buffer having pH 7.4. Cells were then dehydrated using an ethanol gradient, later coated over 200-mesh uncoated copper grids, and observed under STEM (using the Carl Zeiss Sigma 500 Microscope).

#### 2.9.3 Fluorescence bioimaging

To get the fluorescence imaging, the Dewinter fluorescence microscope was used. The cells were seeded in a 60 mm plate and let be confluent to 75–80%. After that, cells were placed on a coverslip, followed by adding DMEM media and incubated for 24 h. The samples to be used for bioimaging were kept under UV light in UV laminar for 30 minutes. Then, 100 μl of the insulin metal nanoparticles (ICoNPs) was taken from each of the three different concentrations described above for MTT assay and added to the respective coverslip containing HEKa cell line opted for the experiment that is, human epidermal keratinocytes, adults (HEKa). In order to get rid of any impurities, each coverslip was washed with PBS buffer twice, followed by cell fixation by adding 2% of formaldehyde solution. Later on, the images were taken using the fluorescence microscope once the cells were fixed to check the role of insulin-protected cobalt core–shell nanoparticles and the effect of different concentrations of samples on fluorescence intensity.

#### 2.9.4 Effect of ICoNPs on recovery of the normal and diabetic wound, *in vitro*, using phase contrast imaging

To determine the effect of prepared nanoparticles on wound healing *in vitro*, the HEKa cells were seeded in 60 mm plates in the presence of glucose (360 mg dl^−1^) for diabetic wounds and in the absence of glucose for normal damages along with DMEM-F12 media (FBS-free medium). They were kept in an incubator at 37 °C and 5% CO_2_ level. The cells were allowed to grow and be 80–85% confluent. Once the plates became confluent, the cell scratch method was used to analyze the effect on healing. In this, the wound is created using a sharp object, that is, the sterile 200 μl tip, and was treated with different concentrations of ICoNPs, insulin, CoCl_2_ salt solution, and the mixture of insulin and CoCl_2_ salt (I + Co). Time-lapse imaging was done to find the change in wound diameter, and the variation in the wound width was measured after 6 h, 12 h, and 24 h, respectively. We randomly measured wound width at different positions for each scratch made in an individual well plate. We took the mean of those independent readings of wound diameter to calculate the percentage change in wound diameter for both the normal and diabetic wounds separately.

### 2.10 Determination of combination index (CI) for cobalt–insulin

The quantitative measure that provides the effect of a single drug in combination with another drug is Combination Index (CI). The level of synergism or antagonism is quantified by estimating the drug combination index in investigating synergistic or antagonistic drug combinations. The combination index of less than 1 (CI < 1) indicates that when different drugs are administered together, they work together to enhance each other's activity, called the synergistic effect. CI values equal to 1 (CI = 1) show that one drug does not interfere with the action of the other hence additive effect, and CI values greater than 1 (CI > 1) indicates the inhibition of drug action by the other drug, thus called the antagonistic effect. For the calculation of the combination index, the cell viability of HEKa cells was determined at varying concentrations of cobalt and insulin, and then calculations were done using eqn (e).

e CI = (D)1/(Dx)1 + (D)2/(Dx)2

where,

f Dx = D*m*[fa/fu]^1/*m*^

Here, (D)1 and (D)2 denote the concentration of cobalt salt and insulin, respectively. The single drug concentrations giving the same effect (Dx)1 and (Dx)2 are determined using the median effect eqn (f). The affected and unaffected cell fractions in the median dose are denoted by fa and fu and are equal to 10^(*y*-intercept)/*m*^, where *m* represents the slope median in the median effect plot of log(*D*) *vs.* log(fa/fu).^26,65,66^

### 2.11 Statistical analysis

The data here is presented as the mean ± SD of at least three independent experiments. Using the one-way ANOVA, the statistical data analysis was done using MS Excel, and the corresponding *p*-value was calculated to check if the data was statistically significant.

## 3. Results and discussions

### 3.1 Structure, composition, and stoichiometry of metal insulin nanoparticles

**Fig. 1:**
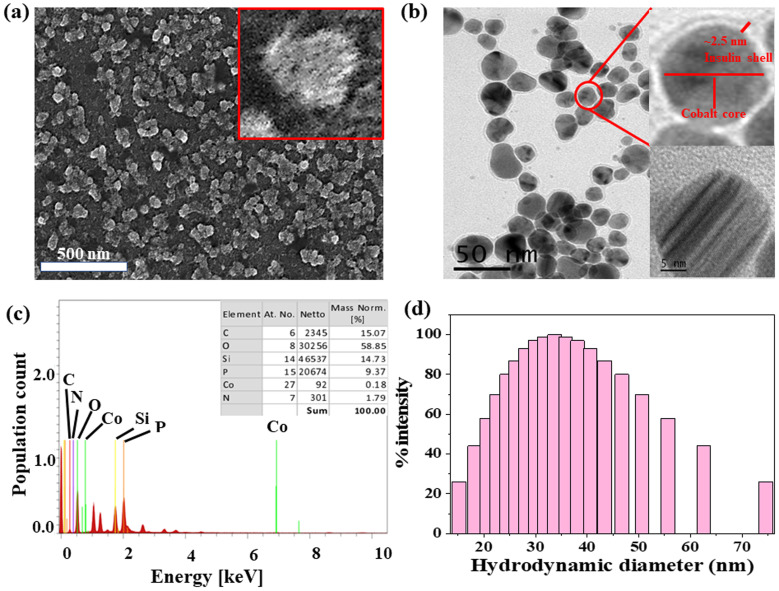
Morphological characterization of ICoNPs (a) SEM micrographs indicating the nanoparticles to be around 10–15 nm at a scale of 100 nm. (b)TEM micrographs of ICoNPs show the nanoparticle size to be ∼13 nm at a scale of 50 nm (inset shows particle size at a scale of 5 nm) (c) EDS showing the presence of cobalt ions in the synthesized nanoparticles (inset shows the relative % of each atom present in the sample) (d) DLS spectra showing the hydrodynamic diameter of ICoNPs *versus* the % intensity of particles.

The formation of spherical nanoparticles of ICoNPs having size 13 ± 2 nm was confirmed by scanning electron microscopic images (at a scale of 500 nm) as shown in Fig. 1a (inset shows the magnified image of core–shell particle which shows the rough surface of nanoparticle due to an outer insulin shell) and transmission electron microscopic images (at a scale of 50 nm) as shown in Fig. 1b and inset of Fig. 1b (at a scale of 5 nm to show the lattice fringes and the insulin coating of ∼2.5 nm around the cobalt core). The nanoparticles are widely distributed over the protein matrix. The presence of different elements like Co, C, O, N, P, and Si is shown in the energy-dispersive X-ray spectroscopy. The even distribution and percentage of Co in the particles come out to be 0.18% as analyzed by EDS studies as indicated in Fig. 1c. The hydrodynamic size of ICoNPs was found to be 35 ± 5 nm as shown in Fig. 1d. Thereafter, the stoichiometric ratio between the insulin protein and Co^2+^ ions was calculated. It was found that ∼3792 insulin proteins encapsulate one nanoparticle. The detailed calculation is provided in ESI.†

### 3.2 *In silico* studies to monitor the interactions between insulin and cobalt ions using metal-ion binding residue templates

**Fig. 2:**
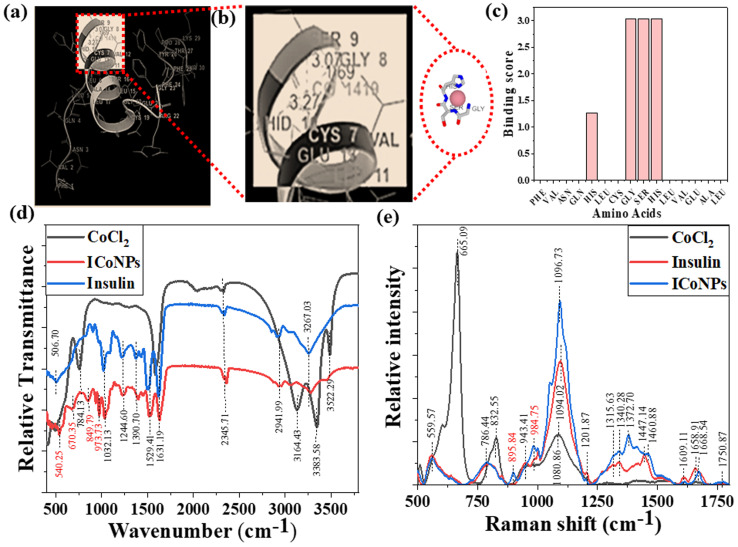
Study for the structural variations in protein due to insulin–cobalt metal ion interactions to form ICoNPs. (a) Metal ion binding residues depict the interaction between chain B of insulin and cobalt ions. (b) The binding sites for cobalt ions at insulin protein with different amino acids within 3.55 Å distance along with the distance measured. (c) The binding score of metal ions with different amino acids in the protein chain (d) FTIR spectra of CoCl_2_, insulin, and ICoNPs in the range 400–4000 cm^−1^ indicate the formation of new bonds between Co–O (vibrations and stretching), CoO–OH bond, and Co–OH bonds which were otherwise not present. (e) Raman spectra of CoCl_2_, insulin, and ICoNPs indicate the involvement of different functional groups of insulin in synthesizing ICoNPs.

An online docking server determined the interaction sites between protein and metal ions, MIB (Metal Ion Binding). With this tool's help, we found the binding sites on chain B of insulin with which the transition metal Co^2+^ can bind. The interaction of the protein with metals is based mainly on the structure and sequence of the amino acids in a protein. Firstly, the sequence of human insulin was extracted from the Protein Data Base (PDB ID: 4EWW) and then inserted in the MIB (Metal Ion Binding) tool to dock the Co^2+^ ions with both the chains (chain A & B) of insulin. As a result, a binding score was assigned to each residue in the insulin protein. If the binding score exceeds the threshold, that particular residue is considered the binding site for that specific metal ion. Therefore, the binding sites for metal ions Co^2+^ were determined. The data indicates that chain A does not show any template indicating the absence of binding sites of Co^2+^ ions with chain A, whereas, in chain B, a binding site was found for Co^2+^ ions. Then we worked with another software called Maestro. Using this, we calculated the distance between the metal ion and its binding site, that is, amino acids, namely 8 GLY, 9 SER, and 10 HIS, present on chain B of insulin. The distance was found to be 1.69, 3.07, and 3.27, respectively. The binding score was also determined using MIB with different amino acids in chain B of insulin with Co^2+^ ions, thus finding the best possible sites for attachment. The binding of cobalt ions with different amino acids is determined and visualized through different orientations of the metal-binding amino acids in proteins. The possible binding sites of cobalt ions with different amino acids in insulin are shown in Fig. 2a and b, the binding score of amino acids with insulin protein is shown in Fig. 2c, and the binding score values are given in Table S1.†

### 3.3 FTIR, Raman spectra, and CD spectroscopy for the interactions between protein and metal ions after the formation of nanoparticles

To figure out the interactions involved in metal–protein binding, FTIR was used for CoCl_2_, insulin, and ICoNPs. The peaks appeared due to intermolecular interactions of insulin and CoCl_2_ at different wavenumbers. Firstly, the peak appeared at 506.70 cm^−1^, which indicates the S–S stretching in insulin and ICoNPs.^32^ Then a peak at 540.25 cm^−1^ in ICoNPs indicates Co–O vibrations.^67^ In addition to the second peak, a peak at 670.35 cm^−1^ shows the Co–O bending, confirming the interaction between insulin and CoCl_2_.^68^ A peak at 784.13 cm^−1^ in CoCl_2_ shows librational vibrations.^69^ Two other peaks at 849.79 and 973.73 indicate the CoO–OH band and Co–OH bending, respectively, in ICoNPs.^67,70^ C–N stretching was observed in insulin and ICoNPs at 1032.13 cm^−1^.^31^ Amide I, amide II, and amide III are found at 1631.19 cm^−1^, 1529.41 cm^−1^, and 1244.60 cm^−1^, respectively, in insulin and ICoNPs.^31,32,34^ C–H bond was observed in insulin and ICoNPs at 1390.70 cm^−1^.^31^ Deformational scissor water vibrations were observed at 1573.72 cm^−1^ in CoCl_2_ only.^69^ Nitrile stretch was there at 2345.71 cm^−1^ in all three solutions.^34^ Intermolecular O–H stretching peaks at 2941.99 cm^−1^ in insulin and ICoNPs, but the peak was observed at 3164.13 cm^−1^ in CoCl_2_.^31^ A single peak in insulin and nanoparticles indicated amine N–H stretching at 3267.03 cm^−1^, which was absent in the CoCl_2_.^71^ For symmetrical and asymmetrical valence vibrations of water, peaks were observed at 3383.58 cm^−1^ and 3522.29 cm^−1^, both in CoCl_2_ but absent in insulin and ICoNPs,^69^ which is shown in Fig. 2d.

Similarly, Raman spectral analysis was used to study the significant conformational changes in insulin after binding with cobalt ions. The S–S stretch and O–C–N bend were observed at 553.29 cm^−1^ and 786.44 cm^−1^, respectively, in both insulin and ICoNPs.^32,72^ The CoO–OH and Co–OH bond was observed at 895.84 cm^−1^ and 984.75 cm^−1^, respectively, only in ICoNPs but was absent in insulin.^67,70^ A weak C–N stretch was observed at 1094.02 cm^−1^ in insulin and 1096.73 cm^−1^.^32^ The C–H bend was found in insulin at 1343.21 cm^−1^ and in ICoNPs at 1372.70 cm^−1^.^32^ The peak that is responsible for amide-III random coils shifts slightly from 1315.63 cm^−1^ in insulin to 1332.38 cm^−1^ in ICoNPs.^73^ However, no such change was observed for amide-III (α-helix), as the peak appears at 1201.87 cm^−1^ in insulin and ICoNPs.^74^ A slight shift was observed for amide-II from 1447.14 cm^−1^ in insulin to 1460.88 cm^−1^ in ICoNPs.^75^ The peak for amide-I was observed at 1658.91 cm^−1^ and 1668.54 cm^−1^, respectively, in insulin and ICoNPs.^76^ The peak for the C=O stretch remains unchanged and is found at 1750.73 cm^−1^ in insulin and ICoNPs,^32^ and this is shown in Fig. 2e. A comparative data of FTIR and Raman spectra are shown in Table 1.

**Table 1:** The comparative wavenumber values indicated by FTIR (in the range 400–4000 cm^−1^) and Raman spectra (500–1800 cm^−1^), respectively, of insulin, CoCl_2,_ and ICoNPs showing the position of different functional groups present, which coins the interaction amongst insulin and CoCl_2_ salt solution indicating the formation of insulin cobalt core–shell nanoparticles

FTIR					Raman				
Functional groups	CoCl_2_	Insulin	ICoNPs	References	Functional group	CoCl_2_	Insulin	ICoNPs	References
S–S stretch	—	506.70	506.70	32	C=O stretch	—	1750.73	1750.73	32
Co–O vibrations	—	—	540.25	67	Amide I	—	1658.91	1668.54	76
Co–O stretching	—	—	670.35	68	Amide II	—	1447.14	1460.88	75
Librational vibrations	784.13	—	—	69	Amide III (α – helix)	—	1201.87	1201.87	74
CoO–OH band	—	—	849.79	67	Amide III (random coils)	—	1315.63	1332.38	73
Co–OH bending	—	—	973.73	70	C–H bend	—	1343.21	1372.70	32
C–N stretching	—	1032.13	1032.13	31	C–N (weak stretch)	—	1094.02	1096.73	32
Amide III	—	1244.60	1244.60	31	Co–OH	1080.66	—	984.75	70
C–H bond	—	1390.70	1390.70	31	CoO–OH	832.55	—	895.84	67
Amide II C=O stretch	—	1529.41	1529.41	34	O–C–N (bend)	—	786.44	786.44	32
Deformational scissor vibration of water	1573.72	—	—	69	S–S stretch	665.09	553.29	553.29	68
Amide I	—	1631.19	1631.19	32					
Nitrile stretch	2345.71	2345.71	2345.71	34					
O–H intermolecular stretching	3164.13	2941.99	2941.99	31					
Amine N–H stretch	—	3267.03	3267.03	71					
Symmetrical and asymmetrical valence vibrations of water	3383.58	—	—	69					
	3522.29								

**Fig. 3:**
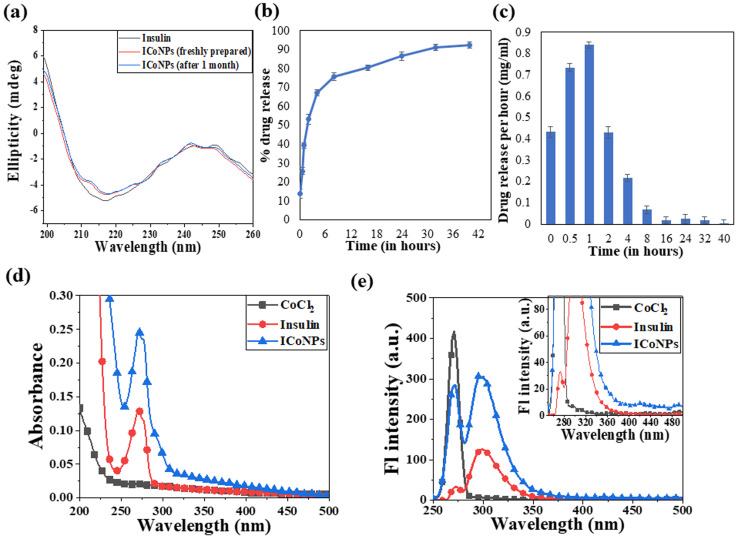
CD spectroscopy, release kinetics, and physical interaction studies of ICoNPs. (a) Circular dichroism spectroscopy studies showing the stability of insulin protein alone and after interaction with cobalt chloride (ICoNPs) (b) the plot shows the release kinetic studies to determine the % drug release from the ICoNPs (c) shows the drug release per hour in mg ml^−1^ analysis of the specificity of interaction of insulin and CoCl_2_ using (d) UV-visible absorption spectra of CoCl_2_, insulin, and ICoNPs showing the peak of insulin alone and after interaction with cobalt (ICoNPs) at ∼272 nm (e) emission spectra of ICoNPs after excitation at 272 nm indicates the emission maxima at ∼300 nm. Inset shows the trailing of fluorescence from ∼420 nm onwards.

Circular dichroism was performed using insulin protein and ICoNPs (freshly prepared and after 1 month) to monitor the interactions between protein and salt solution and find the stability of the secondary structure of protein after binding with cobalt chloride. The three significant far UV signals were recorded in both the solutions, including a positive peak at ∼200 nm and two negative peaks at ∼208 nm (α-helix) and 218 nm (β sheet), which were indicative of the secondary protein structure. The % variation in the peak at 200 nm is 5.23% and 6.21%, at 208 nm is 3.56% and 4.24% and at 218 nm is 4.82% and 5.71% in freshly prepared ICoNPs and ICoNPs after 1 month; respectively and confirmed the protein stability with no significant structural changes were observed, as shown in Fig. 3a.

### 3.4 Drug loading and release kinetic studies

The drug loading capacity and release kinetics of ICoNPs were studied, and the encapsulation efficiency of insulin over the cobalt nanoparticles was found to be 89.48 ± 0.049%. The release kinetic studies were performed at physiological conditions that are pH of 7.4 and a physiological temperature of 37 °C. Burst drug release was observed for the initial 8 h, followed by a sustained drug release afterward. Most of the drug was released by the 40 h. The particles were suspended in a dialysis membrane with a concentration of 3.47 mg ml^−1^, and the OD values were observed at 595 nm after particular intervals of time. It was found that 92.21 ± 0.03% of the drug was released from ICoNPs by the end of 40 hours, making it an efficient drug delivery system in wound healing applications and is shown in Fig. 3b and c. For further confirmation of the release kinetics of the particles, absorbance value was taken at 272 nm (tryptophan absorbance) for all the samples after specific time intervals which shows the gradual increase in OD values with increasing time, indicating the sustained release of insulin from particles and is shown in Fig. S1a.†

### 3.5 Spectroscopic changes after synthesis of core–shell nanoparticles using absorbance and fluorescence spectra

After synthesizing ICoNPs, its spectroscopic characterization was done to observe the absorption and fluorescence intensity of the formed nanoparticles. We obtained a sharp peak for insulin without any added metal salts at 271.61 with an absorbance value of 0.129, whereas, after incubating the insulin with metal salts for 48 h, an absorbance value of 0.245 was obtained for nanoparticles having a peak at 271.50 nm. This confirms the synthesis of ICoNPs, as shown in Fig. 3d. The absorption peak is also compared with tyrosine, as it was selected as a standard and demonstrated in Fig. S1b.†

The ICoNPs were then excited at an excitation wavelength of 270 nm to determine the fluorescence intensity. An emission peak ranging from 280–440 nm was obtained with maxima at ∼303 nm with an intensity of 304.89 a.u when monitored from 200–800 nm, as shown in Fig. 3e. The % change in intrinsic fluorescence intensity was found to be 58.88%. Further, to determine the quantum yield of ICoNPs, tyrosine was used as a standard as its quantum yield value is known. The quantum yield of insulin was found to be 0.1798, and that of ICoNPs turned out to be 0.6825, indicating the fluorescent behavior of formulations synthesized and is shown in Fig. S1c.†

### 3.6 *In vitro* cell studies

#### 3.6.1 HEKa cell viability assay

**Fig. 4:**
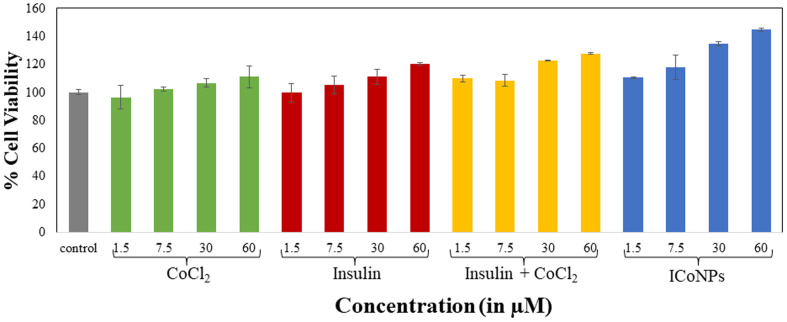
MTT assay to determine the viability and growth rate of HEKa cells. The data shows the treatment of HEKa cells in comparison to the cells treated with varying concentrations (1.5, 7.5, 30, and 60 μM, respectively) of cobalt chloride, insulin, the mixture of insulin and cobalt chloride, and ICoNPs. The data were plotted as the mean value of three independent experiments.

A cell viability test was done to check whether the samples could be employed for biological purposes, which further depends on the mitochondrial activity of cells. Testing was done for insulin, CoCl_2_, a mixture of insulin and CoCl_2,_ and ICoNPs using 1.5, 7.5, 30, and 60 μM of concentrations, and the graph was plotted. The cell viability of untreated cells (control) is taken as 100, and the rest of the studies are compared to this. For CoCl_2_ treated cells, cell viability comes out to be 96.54 ± 6.43%, 102.12 ± 6.36%, 106.75 ± 5.58%, and 110.99 ± 0.77% for 1.5, 7.5, 30, and 60 μM respectively. Insulin shows 99.65 ± 8.14% for 1.5 μM, 105.02 ± 1.48% for 7.5 μM, 110.99 ± 2.89% for 30 μM and 120.26 ± 7.70% for 60 μM respectively. The cells exposed to a combination of insulin and CoCl_2_ showed more cell viability. It is 109.97 ± 2.30%, 108.32 ± 4.17%, 122.78 ± 0.28%, and 127.57 ± 0.56%, respectively, for 1.5, 7.5, 30, and 60 μM respectively. Cell viability after treatment with core–shell ICoNPs showed much more significant changes, which are 110.44 ± 0.70% for 1.5 μM, 117.83 ± 8.76% for 7.5 μM, 134.72 ± 1.76% for 30 μM and 144.69 ± 0.98% for 60 μM respectively and comparative data is shown in Table 2. The graph demonstrated that none of all four samples is toxic to the cells when used in the above-mentioned concentrations. Moreover, with increasing concentration of samples added, the cell viability enhanced (shown in Fig. 4) and was maximum for core–shell ICoNPs formed, indicating that nanoparticles formed are not at all toxic; instead, they help in the cell division and multiplication and thus can be used for wound healing.

The table shows the % variation in mitochondrial reductase activity in the MTT assay for determining cellular metabolism rate using HEKa cells. The cells were treated with varying concentrations of control (CoCl_2,_ insulin, mixture of CoCl_2_ and insulin) and ICoNPs, that is, 1.5 μM, 7.5 μM, 30 μM, and 60 μM respectively, for a duration of 24 hours. The data were plotted as mean value ± SD of three independent experiments. The combination index (CI) values for the cell viability for varying combinations of cobalt salt with insulin protein were calculated to check if the two drugs are synergistic or antagonistic, and the values come out to be less than 1, indicating the synergistic effect of drugs

**Table:** 

% Of change in mitochondrial reductase activity				
Dose	CoCl_2_	Insulin	Insulin + CoCl_2_	ICoNPs
1.5 μM	96.54 ± 6.43	99.65 ± 8.14	109.97 ± 2.30	110.44 ± 0.70
7.5 μM	102.12 ± 6.36	105.02 ± 1.48	108.32 ± 4.17	117.83 ± 8.76
30 μM	106.75 ± 5.58	110.99 ± 2.89	122.78 ± 0.28	134.72 ± 1.76
60 μM	110.99 ± 0.77	120.26 ± 7.70	127.57 ± 0.56	144.69 ± 0.98

**Table:** 

Combination index (CI)			
The concentration of Co salt and insulin	(Dx)1 (Co salt) = D*m*[fa/fu]^1/*m*^	(Dx)2 (insulin) = D*m* [fa/fu]^1/*m*^	CI = (D)1/(Dx)1 + (D)2/(Dx)2
1.5 μM	93 118.86	171 484.6	0.0000265
7.5 μM	71 757.25	128 844.6	0.00018188
30 μM	50 284.49	96 861.06	0.00098882
60 μM	29 936.43	65 800.79	0.00329352

#### 3.6.2 STEM analysis for cellular internalization of ICoNPs

**Fig. 5:**
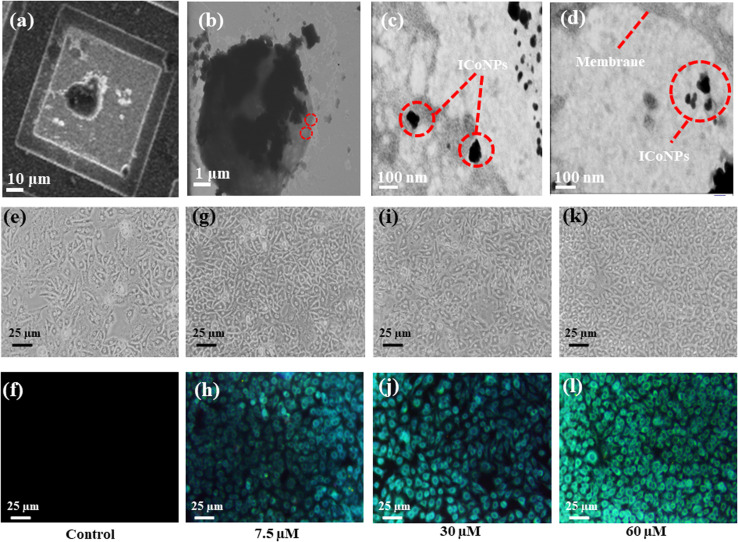
*In vitro* studies indicate nanoparticles' internalization into the cells and their role in bioimaging. (a and b) STEM analysis of cells after incubation with ICoNPs at different scales using a copper grid. (c) Interaction of ICoNPs with the cell membrane receptor leading to its movement inside the lipid bilayer (d) the presence of ICoNPs inside the cell membrane of HEKa cell when observed under a microscope indicating its internalization. The bioimaging of HEKa cells using varying concentrations of ICoNPs. (e) and (f) Shows HEKa cells without any treatment with ICoNPs in white and violet light, respectively. (g) and (h) Indicates the cells treated with 7.5 μM of ICoNPs; (i) and (j) show the cells after treatment with 30 μM of ICoNPs; (k) and (l) indicates the cells after treating them with 60 μM of ICoNPs at white and violet light respectively after a time duration of 3 hours which emits bright blue fluorescence.

STEM analysis was performed after incubating the cells with samples to confirm the interaction and internalization of ICoNPs into the skin cells, followed by their fixation and dehydration by ethanol gradient. The cells were observed under the microscope on copper grids. The mechanism behind cellular internalization can be explained as follows. The synthesized nanoparticles comprise the core–shell structure with cobalt as the core covered by an outer insulin shell. This insulin layer is responsible for the interaction of nanoparticles with human cells. The plasma membrane of target cells in the human body has insulin receptors to which insulin binds and exerts the physiological effect. Insulin receptors have two insulin binding sites that exhibit negative co-operativity; that is, insulin binding to one place prevents its binding to the other and keeps them active throughout. Once the particles bind to the receptors, they can enter the cell membrane's lipid bilayer by following energy-independent direct transduction. Moreover, due to the structural similarity between insulin and IGF-I, it can also bind to IGF receptors and show anti-inflammatory activity. After entering the cell, insulin plays a crucial role in wound healing by controlling distinct mechanisms, including glucose metabolism, protein biosynthesis, and lipid biosynthesis. The interaction of ICoNPs with cellular receptors and their internalization into the cell membrane is shown in Fig. 5a–d (at different scales).

#### 3.6.3 Fluorescence microscope bioimaging

To confirm the broad applications of core–shell insulin–cobalt nanoparticles for cellular imaging, bioimaging was performed on HEKa cell lines. Cells were incubated with particles and showed bright blue fluorescence due to the binding of ICoNPs with the insulin receptors present on the cell wall of each cell, and the fluorescence further goes on increasing with an increase in the concentration of ICoNPs. The imaging was performed on both white and violet light. The control cells without treatment with core–shell ICoNPs are shown in Fig. 5e in white light and Fig. 5f in violet light, indicating no fluorescence in cells. Fig. 5g, i, and k show the cells under white light, and the Fig. 5h, j, and l show the cells in violet light after treatment with varying concentrations of ICoNPs, that is, 7.5 μM, 30 μM, and 60 μM respectively. It suggests the nanoparticles illuminate the cell walls with bright blue color; therefore, they can be used for bioimaging to monitor the changes inside the cells during the entire healing process.

#### 3.6.4 HEKa cell migration assay in diabetic and normal wound conditions

**Fig. 6:**
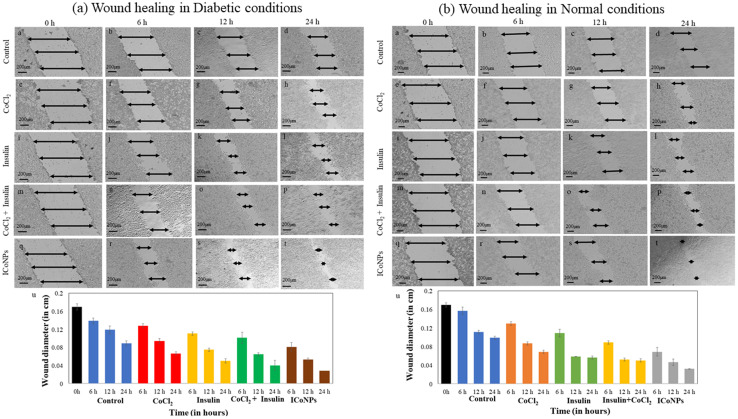
Promotion and monitoring of *in vitro* wound recovery of the diabetic and normal wound using ICoNPs. The nanoparticles induced better wound recovery in HEKa cells when compared with cobalt salt, insulin, or a mixture of both. The cells were incubated with a fixed concentration of all the solutions, that is, 30 μM. HEKa cells were taken as control (without any added formulations). (a) The figure shows diabetic wound healing using HEKa cells (a) 0 h, (b) 6 h, (c) 12 h, (d) 24 h; the cells treated with the salt solution of cobalt chloride after (e) 0 h, (f) 6 h, (g) 12 h, (h) 24 h; the HEKa cells after treatment with insulin protein (i) 0 h, (j) 6 h, (k) 12 h, (l) 24 h; the cells after treating them with a mixture of insulin and cobalt chloride are shown in figure (m) 0 h, (n) 6 h, (o) 12 h, (p) 24 h; cells after treatment with ICoNPs after a duration of (q) 0 h, (r) 6 h, (s) 12 h, (t) 24 h; respectively. (u) The plot shows the relative change in wound diameter in diabetic conditions after treatment with all the solutions respectively after specific time intervals. (b) The figure shows normal wound healing using HEKa cells (a) 0 h, (b) 6 h, (c) 12 h, (d) 24 h; the cells treated with the salt solution of cobalt chloride after (e) 0 h, (f) 6 h (g) 12 h (h) 24 h; the HEKa cells after treatment with insulin protein (i) 0 h (j) 6 h (k) 12 h (l) 24 h; the cells after treating them with a mixture of insulin and cobalt chloride are shown in figure (m) 0 h (n) 6 h (o) 12 h (p) 24 h; cells after treatment with ICoNPs after a duration of (q) 0 h (r) 6 h (s) 12 h (t) 24 h respectively. (u) The plot shows the relative change in wound diameter in normal conditions after treatment with all the solutions respectively after specific time intervals.

The core–shell metal protein nanoparticles induce cell migration after treatment with CoCl_2_, insulin, insulin + CoCl_2_ mixture, and ICoNPs in normal and diabetic wounds. However, the increase is more in normal injuries than in diabetic wounds. With increasing time, the extent of cell division and migration increased when monitored at a fixed concentration of 30 μM. For the change in wound diameter, we measured wound width at three distinguished positions for each scratch made on an individual plate. We took the mean of those independent readings of wound diameter to calculate the percentage change in wound diameter with time. The change in wound diameter of HEKa cells taken as control after 0, 6, 12, and 24 h is indicated in Fig. 6(a)a–d. In diabetic conditions, the cells treated with core–shell ICoNPs show the percentage change of the scratched wound after 6, 12, and 24 h as 41.4 ± 1.08%, 54.94 ± 0.97%, and 67.66 ± 0.28%, respectively, and is shown in Fig. 6(a)q–t. A similar procedure was followed, and the % of a gap left in cells that were treated with CoCl_2_, insulin, and insulin + CoCl_2_ was calculated and showed significant migration when compared to control but less than that of core–shell ICoNPs. The % change in a migration after 6, 12, and 24 h is noticed in all samples and came out to be 27.12 ± 0.50%, 45.58 ± 1.26%, and 54.45 ± 0.35% in cells incubated with a mixture of insulin + CoCl_2_ (Fig. 6(a)m–p). In cells treated with insulin, the value of change in migration is 20.27 ± 0.35%, 37.08 ± 0.35%, and 43.29 ± 0.33%, respectively (Fig. 6(a)i–l), while it is 7.88 ± 0.60%, 21.09 ± 0.35% and 24.83 ± 0.70% in cells treated with CoCl_2_ (Fig. 6(a)e–h). A comparative study of variation in scratch diameter of the diabetic wound after treating the cells with all four samples is shown graphically in Fig. 6(a)u.

Similarly, in the normal wound, the variation in scratch diameter for control cells is shown in Fig. 6(b)a–d. The % change in wound diameter is monitored after the same time interval in normal conditions and found to be 14.84 ± 0.60%, 31.75 ± 0.35%, and 34.7 ± 0.70% in the cells treated with CoCl_2_ (Fig. 6(b)e–h). In the cells treated with insulin, insulin + CoCl_2,_ the % variation in migration with time is more as compared to CoCl_2_ and is equal to 30.33 ± 0.80%, 41.27 ± 0.70%, and 48.98 ± 0.35% in insulin (Fig. 6(b)i–l), 40.65 ± 0.35%, 50.8 ± 0.35% and 59.19 ± 0.35% in insulin + CoCl_2_ (Fig. 6(b)m–p). The core–shell ICoNPs show the maximum migration, and the % wound diameter changed is 56.13 ± 0.92%, 63.50 ± 0.70%, and 71.43 ± 0.35%, respectively, indicating the significance of prepared nanoparticles in the healing application of normal wounds (Fig. 6(b)q–t). The graph in Fig. 6(b)u shows the variation in normal wound diameter as a comparative study of all the formulations after particular time intervals. A comparative data table of change in wound migration in normal and diabetic wounds is shown in Table S2.† The statistical significance of data was determined by calculating *p* values for the scratch assay in diabetic and normal wound conditions using one-way ANOVA. The comparative data are presented in Table S3.†

### 3.7 Combination index of cobalt and insulin

To calculate CI, D*m* was calculated using *m* and *y* from Fig. S2a† for cobalt and Fig. S2b† for insulin. It was found from the CI's calculated values that the cobalt chloride salt and insulin show a synergistic effect. The values calculated for varying combinations of insulin and cobalt chloride concentrations come out to be less than one indicating the synergism between the two and, thus, work together to enhance each other's activity. This is in accordance with the results obtained for cell viability and cell migration assay indicating the significant role of insulin cobalt nanoparticles in faster wound healing applications. The data is shown in Table 2.

## 4. Conclusion

The wounds were sometimes reopened to maintain the proper healing environment and eliminate any complications in the wound recovery process, paving the way for improper healing, enhanced healthcare costs, and decreased life quality. Thus, suitable agents are always required, which assist in selectively binding to the wound site and guide in monitoring the progress of wound recovery. We formulated novel core–shell ICoNPs containing insulin and cobalt and evaluated their role in wound healing activity. Insulin promotes cellular growth by modulating inflammation, reduces blood glucose concentration, and exhibits anti-inflammatory activity. Cobalt is known to reduce the microbial contamination load. Thus, the formulations were checked for a synergistic effect in wound treatment. Different techniques were used to confirm their formation, including TEM micrography, elemental analysis, FTIR, Raman spectroscopy, and metal ion binding site prediction and docking server (MIB) to ensure Co–metal interaction with the insulin protein. From FTIR and Raman spectra, we got peaks showing the intermolecular interaction between Co and insulin and exhibiting moderate structural changes in amide regions of the protein. The metal–ion binding site prediction and docking server were used to find possible sites for the metal ion to bind with insulin amino acid chains A and B. No site was observed in chain A, while in chain B, there was a binding site for Co^2+^ ions, and the amino acids involved in groove formation were 8 GLY, 9 SER, and 10 HIS. The loading efficiency and release kinetic studies were done to determine the effective loading of insulin drugs over the nanoparticles. The synthesized core–shell nanoparticles were tested for their effect on cell viability to be used further for diabetic and normal wound healing and bioimaging applications. The calculated values for the MTT assay and combination index values indicate the synergism between the cobalt chloride salt and insulin protein, making them suitable for further utilization in biological activities. The formed core–shell nanoparticles, ICoNPs, show excellent target-specificity. Due to the insulin consumption in their synthesis, they exhibit the property of binding to insulin receptors, making these particles unique from other particles in various respects, mainly in target-specificity properties. Further, they have enhanced fluorescent properties when compared with insulin alone which can be easily detected using fluorescence microscopy and fluorescence bioimaging. They also possess an enormous tendency to heal both diabetic and normal wounds. Thus, it has a wide range of applications in healing, bioimaging, and material and biomaterial sciences. Therefore, it can be further explored for *in vivo* applications in diverse fields.

## Conflicts of interest

Authors declare no conflict of interest.

## Supplementary Material

RA-013-D3RA01473H-s001
