# Supplementary material for: Insulin–cobalt core–shell nanoparticles for receptor-targeted bioimaging and diabetic wound healing
Source: RSC Adv. 2023 Jul 6;13(29):20321–35. doi: 10.1039/d3ra01473h (PMC10323873; doi:10.1039/d3ra01473h)
Supplement: RA-013-D3RA01473H-s001 [file RA-013-D3RA01473H-s001.pdf]

Electronic Supplementary Material (ESI) for RSC Advances.
This journal is © The Royal Society of Chemistry 2023

Insulin-protected cobalt nanoparticles for their receptor-targeted bioimaging and diabetic wound healing

Deepinder Sharda[1], Diptiman Choudhury[1,2]*

1; School of Chemistry and Biochemistry, Thapar Institute of Engineering and Technology, Patiala, 147004, Punjab, India.

2; Thapar Institute of Engineering and Technology-Virginia Tech (USA) Center of Excellence in Emerging Materials, Thapar Institute of Engineering and Technology, Patiala, Punjab-147004, India.

*Corresponding E-mail: diptiman@thapar.edu ; Phone: +91-8196949843

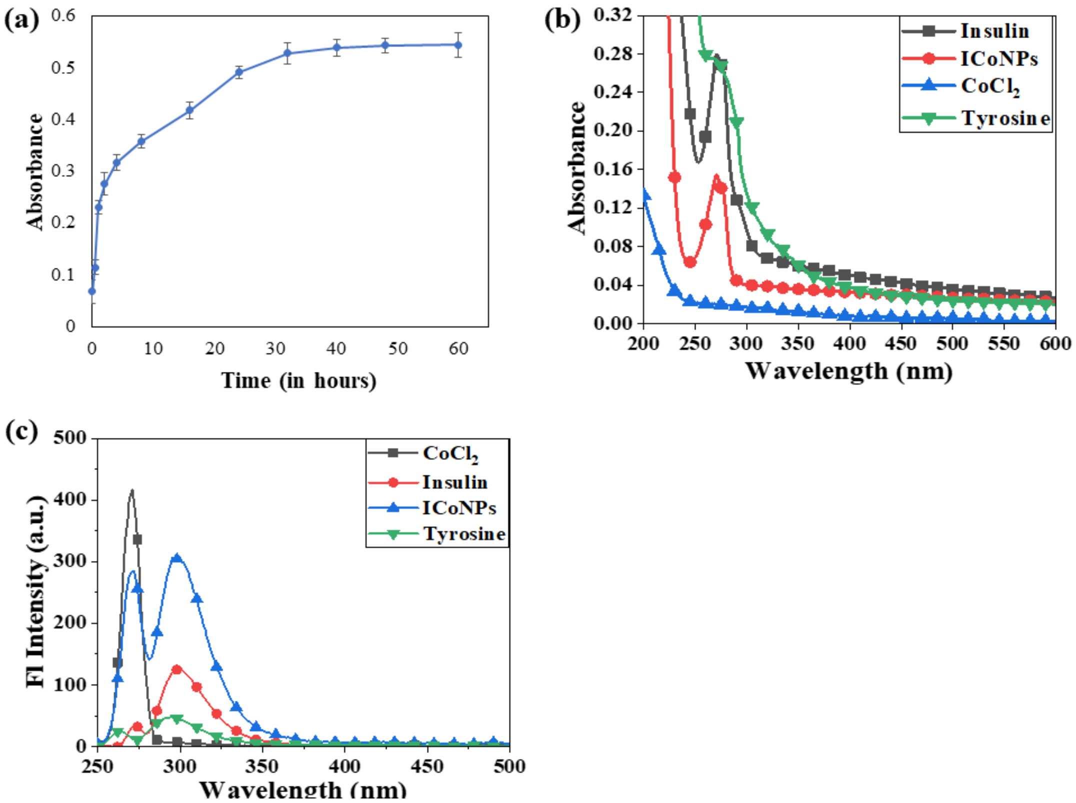

Fig. S1 (a) It shows the release kinetics data when the OD value was taken at 272 nm, which is the excitation value of tryptophan. It indicates a sustained drug release pattern, as was confirmed by using the Bradford assay. (b) It compares absorbance values between Insulin, ICoNPs, $CoCl_2$, and tyrosine (standard). (c) It compares the fluorescence intensity value of Insulin, ICoNPs, $CoCl_2$, and tyrosine (standard) to calculate the quantum yield enhancement in insulin and insulin cobalt core-shell nanoparticles.

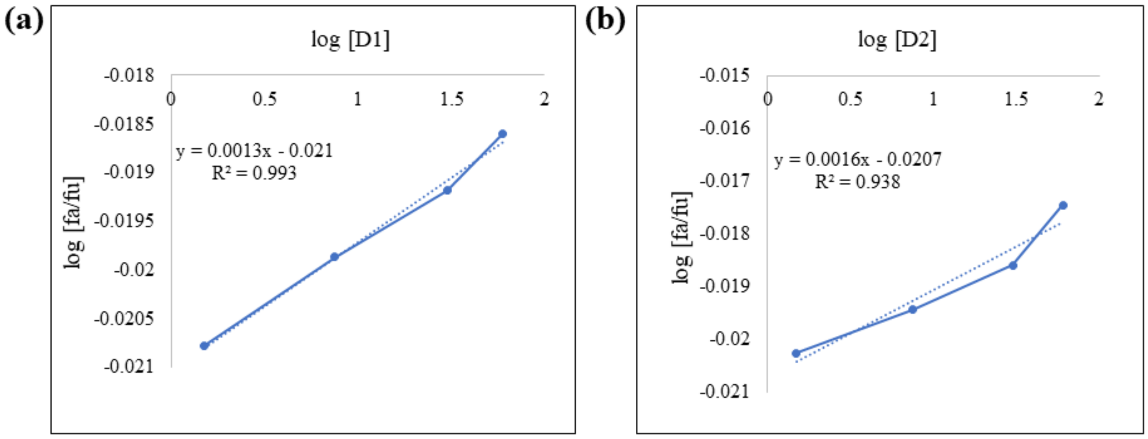

Fig. S2 Median plots of (A) Co salt, (B) Insulin for finding the y-intercept and m values to calculate Dm to determine the combination index of cobalt and insulin.

Table S1. It shows the binding score for different amino acids present in chain B of insulin with Ni^{2+} ion. Here the binding score is maximum for SER and HIS amino acids.

**Residue Number**	**Amino Acid**	**Score**
1	PHE	-0.358
2	VAL	-0.358
3	ASN	-0.358
4	GLN	-0.358
5	HIS	1.270
6	LEU	-0.358
7	CYS	-0.358
8	GLY	3.037
9	SER	3.037
10	HIS	3.037
11	LEU	-0.358
12	VAL	-0.358
13	GLU	-0.358
14	ALA	-0.358
15	LEU	-0.358

Table S2. It shows the comparative data of variation in wound diameter in diabetic and normal conditions after treatment with 30 µM of insulin, cobalt chloride, and the mixture of insulin and cobalt chloride and ICoNPs. The data was measured after a time duration of 6, 12, and 24 h.

% Change in diabetic wound diameter with time				
**Time**	**$CoCl_2$**	**Insulin**	**Insulin +$CoCl_2$**	**ICoNPs**
**6 h**	7.88 ± 0.59 %	20.27 ± 0.35 %	27.12 ± 0.50 %	41.4 ± 1.08 %
**12 h**	21.09 ± 0.47 %	37.08 ± 0.35 %	45.58 ± 1.26 %	54.94 ± 0.97 %
**24 h**	24.83 ± 0.60 %	43.29 ± 0.33 %	54.45 ± 0.35 %	67.66 ± 0.28 %
**% Change in normal wound diameter with time**				
**Time**	**$CoCl_2$**	**Insulin**	**Insulin +$CoCl_2$**	**ICoNPs**
**6 h**	14.84 ± 0.60 %	30.33 ± 0.80 %	40.65 ± 0.35 %	56.13 ± 0.92 %
**12 h**	31.75 ± 0.35 %	41.27 ± 0.70 %	50.8 ± 0.35 %	63.50 ± 0.70 %
**24 h**	34.7 ± 0.70 %	48.98 ± 0.35 %	59.19 ± 0.35 %	71.43 ± 0.35 %

Table S3. It shows the p values calculated for % variation in wound diameter in diabetic and normal conditions after treatment with 30 µM of insulin, cobalt chloride, the mixture of both insulin and cobalt chloride, and ICoNPs. The statistical significance of data is considered when $p < 0.05$.

P value for checking the statistical significance of data for diabetic wound				
**Time**	**$CoCl_2$**	**Insulin**	**Insulin +$CoCl_2$**	**ICoNPs**
**6 h**	0.10824	0.003371	0.001639	0.001567
**12 h**	0.005436	0.000469	0.000585	0.000618
**24 h**	0.023327	0.002122	0.000904	0.00038
**P value for checking the statistical significance of data for normal wound**				
**Time**	**$CoCl_2$**	**Insulin**	**Insulin +$CoCl_2$**	**ICoNPs**
**6 h**	0.005648	0.001751	0.000152	0.000222
**12 h**	0.001058	1.33E-05	2.59E-05	0.000139
**24 h**	0.000447	0.00012	7.07E-05	5.44E-06

The Calculation of stoichiometry between cobalt and insulin protein

The stoichiometry was calculated and found out to be that one cobalt nanoparticles (13 nm diameter) is encapsulated by ~ 3791.66 insulin protein.

Volume of a particle = $4/3\ \pi\ r^3$
Radius = 6.5 nm (13 nm diameter of cobalt nanoparticles from TEM data)
$= 4/3 \times 3.14 \times (6.5 \times 10^{-7}\ cm)^3$
$= 1149.76 \times 10^{-21}\ cm^3$
Atomic radius of Co atom = 200 pm = 2×10^{-8} cm
Volume of Co atom = $4/3 \times 3.14 \times (2 \times 10^{-8}\ cm)^3$
$= 33.4933 \times 10^{-24}\ cm^3$
$V_{nanoparticle} = N \times V_{atom}$
$N = V_{nanoparticle} / V_{atom}$

$= 1149.76 \times 10^{-21}$ cm^3 / 33.493×10^{-24} cm^3
$= 34.328 \times 10^3$
= 34328 number of atoms of cobalt in one nanoparticle
$= 10^6$ µM of Co^{2+} in 10^6µL $= 6.023 \times 10^{23}$
Therefore,
= 1µM of Co^{2+} in 10^6 µL $= 6.023 \times 10^{23}/ 10^6$
= 1µM of Co^{2+} in 1µL $= 6.023 \times 10^{23}/ 10^6 \times 10^6$
= 1.82 µM of Co^{2+} in 227µL $= 227\times 1.82 \times 6.023 \times 10^{23}/ 10^{12}$
$= 2.488 \times 10^{14}$ atoms
Therefore, number of particles in total solution
$= 2488 \times 10^{11}/ 34328$
$= 0.724 \times 10^{12}$
Therefore
2.5 ml will have $= 0.724 \times 10^{12}$ particles
Thus,
1000 ml will have $= 0.724 \times 10^{12} \times 10^3/ 2.5$
$= 0.2896 \times 10^{15}$
$= 0.2896 \times 10^{15}$ number of particles / litre
$= 0.2896 \times 10^{15}/ 6.023 \times 10^{23}$ M
$= 0.48 \times 10^{-9}$ M
= 0.48 nM
Per particle insulin = 1820 nM / 0.48 nM
= 3791.66 ~ 3792
